# Magnetic resonance imaging changes of sacroiliac joints in patients with recent-onset inflammatory back pain: inter-reader reliability and prevalence of abnormalities

**DOI:** 10.1186/ar1859

**Published:** 2005-12-01

**Authors:** Liesbeth Heuft-Dorenbosch, René Weijers, Robert Landewé, Sjef van der Linden, Désirée van der Heijde

**Affiliations:** 1 Department of Internal Medicine, Division of Rheumatology, University Hospital Maastricht, The Netherlands; 2 Department of Radiology, University Hospital Maastricht, The Netherlands; 3 CAPHRI Research Institute, University Maastricht, The Netherlands

## Abstract

To study the inter-reader reliability of detecting abnormalities of sacroiliac (SI) joints in patients with recent-onset inflammatory back pain by magnetic resonance imaging (MRI), and to study the prevalence of inflammation and structural changes at various sites of the SI joints.

Sixty-eight patients with inflammatory back pain (at least four of the five following criteria: symptom onset before age 40, insidious onset, morning stiffness, duration >3 months, improvement with exercise — or three out of five of these plus night pain) were included (38% male; mean age, 34.9 years [standard deviation 10.3]; 46% HLA-B27-positive; mean symptom duration, 18 months), with symptom duration <2 years. A MRI scan of the SI joints was made in the coronal plane with the following sequences: T1-weighted spin echo, short-tau inversion recovery, T2-weighted fast-spin echo with fat saturation, and T1-spin echo with fat saturation after the administration of gadolinium. Both SI joints were scored for inflammation (separately for subchondral bone and bone marrow, joint space, joint capsule, ligaments) as well as for structural changes (erosions, sclerosis, ankylosis), by two observers independently. Agreement between the two readers was analysed by concordance and discordance rates and by kappa statistics.

Inflammation was present in 32 SI joints of 22 patients, most frequently located in bone marrow and/or subchondral bone (29 joints in 21 patients). Readers agreed on the presence of inflammation in 85% of the cases in the right SI joint and in 78% of the cases in the left SI joint. Structural changes on MRI were present in 11 patients. Ten of these 11 patients also showed signs of inflammation.

Agreement on the presence or absence of inflammation and structural changes of SI joints by MRI was acceptable, and was sufficiently high to be useful in ascertaining inflammatory and structural changes due to sacroiliitis. About one-third of patients with recent-onset inflammatory back pain show inflammation, and about one-sixth show structural changes in at least one SI joint.

## Introduction

Ankylosing spondylitis (AS) is a chronic rheumatic condition, characterized by inflammation of the axial skeleton, particularly the sacroiliac (SI) joints. Patients fulfil classification criteria for AS if characteristic radiological changes of the SI joint are present, together with defined clinical symptoms and findings [1]. AS belongs to the group of seronegative spondyloarthritides (SpA). The European Spondylarthropathy Study Group has developed classification criteria for SpA [2].

Sacroiliitis is a characteristic feature of AS and is frequently found in patients with SpA, although it is not obligatory. Patients with sacroiliitis experience chronic low back pain with an inflammatory pattern that often begins in young adulthood. Because chronic low back pain is common in the population, sacroiliitis is often not considered as a cause of back pain. Besides, early sacroiliitis is often not visible on conventional radiographs, or is difficult to interpret, which may lead to a long delay in establishing a diagnosis. Frequently, a mean duration of more than eight years between the start of symptoms and the diagnosis of AS is reported [3,4]. Such a delay is increasingly unwarranted because of the availability of effective treatment. Magnetic resonance imaging (MRI) is an imaging modality that may shorten the delay between the start of symptoms and a classifying diagnosis of AS or SpA since MRI can detect inflammation early [5-7]. Algorithms for diagnostic purposes have recently been proposed in which MRI of the SI joints was attributed a prominent place [8]. In order to judge whether MRI is helpful in making an early diagnosis, the psychometric properties of assessing inflammation and structural changes by MRI should appropriately be tested in patients with very early disease and not only in those with advanced AS. The aim of this study was to evaluate whether MRI could reliably assess inflammation and structural damage of SI joints in patients with short-term inflammatory back pain.

## Methods

### Patients

Patients with inflammatory low back pain present for two years at most, and without a confirmed rheumatologic diagnosis, were eligible for this study. Inflammatory back pain was defined according to the Calin criteria [9]. Inflammatory back pain by these criteria is defined if at least four of the five following characteristics are present: insidious onset, onset before the age of 40 years, persistence for at least three months, association with morning stiffness, and improvement with exercise. Patients also could be included if three out of five of these criteria were present plus night pain. Preferably, but this is not obligatory, patients should have at least one feature of SpA according to the European Spondylarthropathy Study Group criteria: presence of a family member with AS, and presence or history of psoriasis, inflammatory bowel disease (IBD) or uveitis.

The study was approved by the institutional review board and all patients gave written informed consent.

### Magnetic resonance imaging

A MRI examination of the SI joints was performed using a 1.5 Tesla Philips Gyro scan ACS-NT (Philps, Best, The Netherlands). Patients were scanned in a supine position using a Synergy-spine coil as the surface coil. We chose a coronal oblique scan plane parallel to the length of the sacrum and two slabs: one transversal slab was positioned cranially to the region of interest, to diminish flow artefacts; and one was positioned frontally through the bowel and anterior abdominal wall, to diminish motion artefacts of breathing and bowel movements. The following sequences were used: T1-weighted spin echo (SE), short-tau inversion recovery (STIR), T2-weighted fast SE with fat saturation, and T1-weighted SE with fat suppression after the intravenous administration of contrast medium (gadolinium diethylenetriaminepentate, 0.1 mmol/kg body weight).

Different relevant MRI findings with regard to sacroiliitis were identified from the literature; a differentiation was made between inflammatory changes and structural changes and the different localization of these changes. Pathological changes of interest were defined as inflammation and structural changes including erosions, sclerosis and ankylosis. Regions of interest were the subchondral region, the bone marrow, the joint capsule, the joint space and the retro-auricular ligaments.

Firstly, in different sessions, MRI scans were reviewed and scored together by two observers (LHD and RW) and discrepancies in scoring were extensively discussed. After these training sessions, inter-reader reliability was assessed for a small subset of MRI scans. As the reliability appeared sufficiently high, each MRI was thereafter independently scored by these two observers, who were blind for the patient identity and for clinical, laboratory and radiological data. Findings were graded as 0 (absent), 1 (minimal), 2 (moderate) and 3 (extensive). Inflammation was scored per SI joint in the subchondral region (the region adjacent to the cortical lamella, extending 0.5 cm into the bone marrow cavity), the bone marrow, the joint capsule (the transition of the joint space to para-articular soft tissue), the joint space (defined as the space between the cortical lamellae) and the retro-auricular ligaments. Inflammation was defined as a low signal intensity on T1, with enhancement after gadolinium administration, and/or high signal intensity on STIR and/or T2 fast SE. Inflammation in ligaments was defined as areas of low signal intensity running through high signal intensity tissue on T1, which reflects interosseous ligaments crossing juxta-articular fatty tissue.

Structural changes were scored per SI joint, and included erosions (an irregularly delineated joint space on T1), sclerosis (low signal intensity on T1, STIR and T2 fast SE, without enhancement after gadolinium administration) and ankylosis (the disappearance of the joint space in all sequences).

Inflammation and sclerosis were scored on the iliac and sacral side of both SI joints separately. Erosions and ankylosis were scored for the entire left and right SI joints. Active inflammation was defined as inflammation in at least one of the joint regions (subchondral bone, bone marrow, ligaments, joint capsule, joint space) and the presence of structural damage as erosions, sclerosis and/or ankylosis per SI joint.

### Analysis

Agreement between both MRI readers with respect to inflammation (per site) and chronic changes (sclerosis, erosions and ankylosis) was analysed by cross-tabulation, by concordance and discordance rates, and by kappa statistics (unweighted Cohen's kappa).

## Results

### Patients

Of the 70 patients that were selected for the study, two patients were excluded (one because of claustrophobia and one because of withdrawal of consent); therefore, complete data for 68 patients were available for analysis. The characteristics of the patients are presented in Table 1. One-half of the patients were HLA-B27-positive. One-third of the patients reported a history of either psoriasis, IBD or uveitis. Two additional patients reported a history of both uveitis and IBD, and one patient reported psoriasis and IBD. Of the 25 patients (37%) with a family history of AS, four patients had a medical history of uveitis and two patients a history of IBD. Fifteen patients (22%) did not have any of the additional SpA features. Of these 15 patients, seven were HLA-B27-positive.

### Agreement on MRI findings

**Figure 1:**
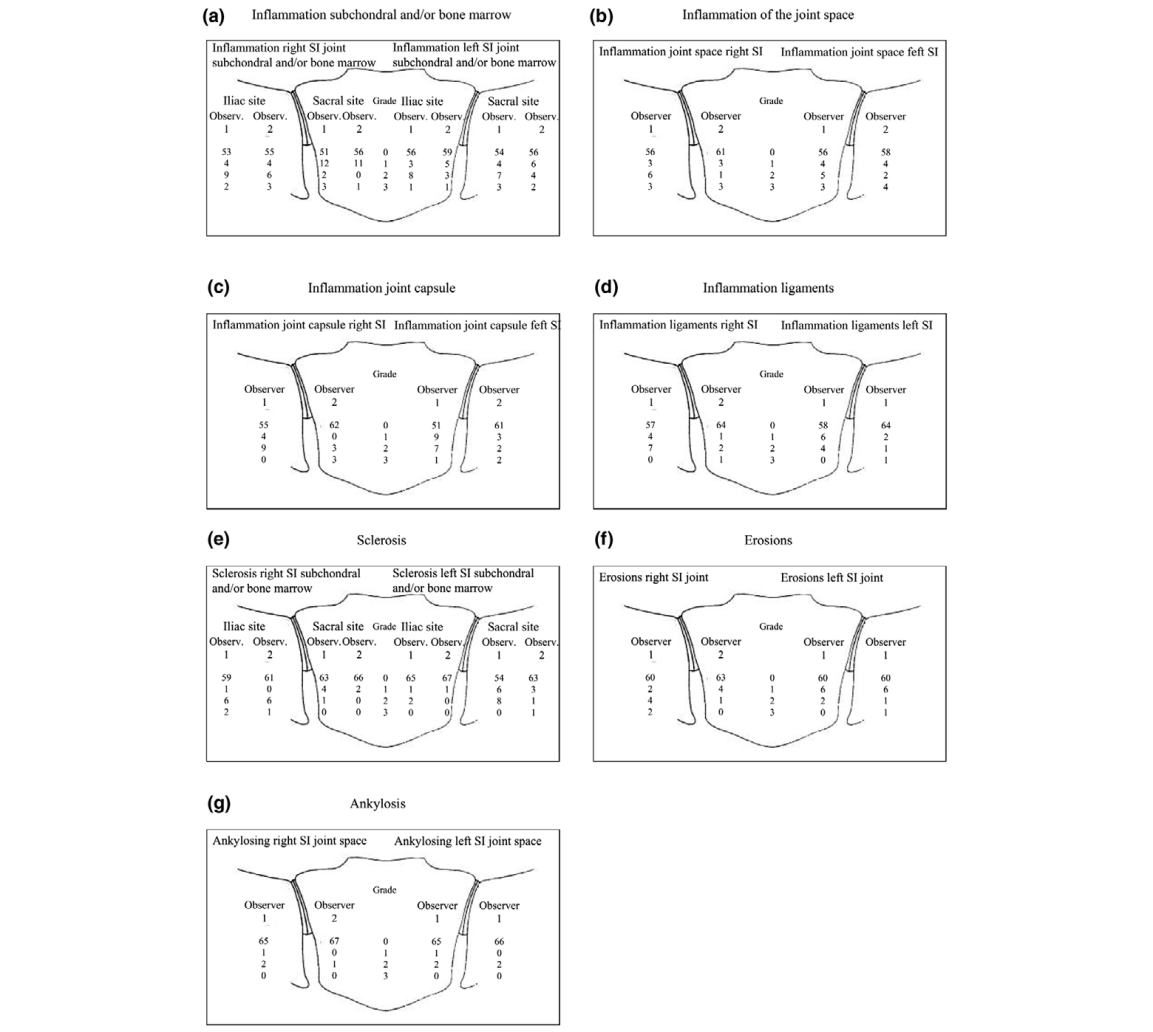
Schematic presentation of the number of pathological findings by the two observers. All abnormalities are graded for extensiveness as 0 (absent), 1 (minimal), 2 (moderate) and 3 (extensive) in the right and left sacroiliac (SI) joints in 68 patients. For inflammation in subchondral bone and bone marrow, and for sclerosis, the data are presented for each site of the joint. For the other abnormalities, the grading for the entire joint per observer is presented.

Figure 1 shows the frequency and localization of inflammation (Figure 1a–d) and chronic changes (sclerosis [Figure 1e], erosions [Figure 1f] and ankylosis [Figure 1g]) per SI joint per observer. Inflammation of the subchondral region and the bone marrow was the most frequently observed finding. It can be seen that both readers use all grades, but pathological findings were mostly scored as grade 2, representing moderate involvement. For the further analyses all positive findings are grouped together irrespective of the grade applied.

Table 2 presents data on the inter-observer agreement with respect to the inflammatory and structural findings. Readers agreed on the presence of inflammation at any site in 85% of the cases in the right SI joint, and in 78% of the cases in the left SI joint. Kappa values reflecting the agreement for detecting inflammation were reasonable (right SI joint, 0.68; left SI joint, 0.51). Table 2 also provides insight into the prevalence of the various findings in this population with early inflammatory back pain. Based on the concordance and discordance rates, agreement is very similar for all assessed sites. However, due to the low prevalence of inflammation at several of the locations and structural changes overall, Cohen's kappa values are influenced negatively. The lowest kappa value and also the lowest concordance rate is found for inflammation of the joint capsule of the left SI joint, which was present in only three patients.

Table 3 summarizes the concordant findings of both readers with respect to inflammation and structural changes on a patient level. Twenty-two out of 68 patients showed any sign of inflammation; 10 patients in both SI joints and 12 patients unilaterally. Only one patient had inflammation excluding the bone marrow and subchondral bone. Ten of these 22 patients with inflammation in one or both joints also had structural changes. Only one patient had structural changes without inflammation. Similarly to inflammation, about one half of the patients with structural changes showed these changes bilaterally (six out of 11).

## Discussion

One of the important aims of this study was to establish whether inflammation and structural changes on MRI could reliably be assessed. In order to allow a detailed judgement, and to trace redundancies, we decided to score inflammation and structural changes per site and per type of lesion. It can be concluded that the agreement between both readers about the presence or absence of pathological findings on MRI was reasonable, especially for inflammation at sites were it was most prevalent. With agreement levels mostly around 85% for the presence of inflammation overall and at different locations, it seems sufficiently high to justify a conclusion of inflammation made by one observer in clinical practice. Expectedly with reference to the population under study, the prevalence of chronic changes on MRI was low. Because of this low prevalence of structural changes, the reliability of scoring these changes is more difficult to assess. This similarly applies to the assessment of inflammation in the joint space, capsule and ligaments. Notwithstanding this limitation, the overall agreement for the different sites of the joint was comparable, with a possible exception for inflammation in the joint capsule. An explanation may be that the delineation of the joint capsule is poorly defined, which may give rise to misinterpretations.

Another important finding in this study was that it is probably sufficient to look for bone marrow oedema and/or subchondral inflammation. The contribution of other sites of the joint to make a diagnosis of inflammation was only marginal. We found only one patient in whom inflammation was restricted to joint capsule and ligaments.

A few studies reported agreement with respect to lesions found on MRI examination of the SI joints, but none of the studies was performed in patients with recent onset inflammatory back pain. Bigot and colleagues proposed 11 criteria referring to both the synovial and the fibrous part of the SI joint that point to sacroiliitis, and showed a good intra-observer and inter-observer reliability (a kappa value of 0.89 for detecting bone marrow oedema) [10]. However, this was a study in 22 SpA patients with established disease, in which grade 2 radiological sacroiliitis according to the New York criteria was present in 80% of the SI joints. Puhakka and colleagues have proposed a scoring system for MRI abnormalities of the SI joints, which distinguished inflammatory activity as well as joint damage [11]. Inter-observer reliability in this study, which included 41 patients with SpA, of whom 20 patients had grade 2 sacroiliitis or more of at least one SI joint on radiography, was importantly lower (a kappa value of 0.47 for bone marrow enhancement, and of 0.67 for joint space enhancement) as compared with our study. Finally, Docherty and colleagues found a kappa value of 0.63 for inter-observer agreement with respect to inflammation on MRI in a study of 20 patients with established or suspected sacroiliitis on radiographs, but contrast administration was not performed [12]. Our results are largely in accordance with the published literature, although comparability is limited due to the differences in study population, with the prevalence of the abnormalities largely influencing kappa values.

In this cohort of inflammatory back pain of less than two years duration, inflammation in the SI joints on MRI could be detected in about one third of the patients (22/68). Moreover, one sixth of patients already showed signs of structural changes on the MRI scan (11/68 patients). Although the number of patients with structural changes is low, this finding indicates that MRI might be a useful tool in the assessment of patients with early inflammatory back pain. It is the amount of variation in the outcome of interest (inflammation and/or structural changes) rather than the number of patients under study that is important in judging whether the sample size is sufficient to test reliability. As long as all kinds of abnormalities are covered, it is possible to test reliability even in situations like this, with only 11 patients showing abnormalities. Undoubtedly, however, the likelihood of covering all kinds of abnormalities will increase by increasing patient number. The real value of MRI will therefore be ascertained in future, by following the patients longitudinally and obtaining more data, which will occur in this cohort. The development and choice of an appropriate scoring system for sacroiliitis on MRI to be used in clinical studies and trials will be subject of interest in an ongoing ASAS-OMERACT working group [13].

## Conclusion

MRI can reliably detect inflammation and structural changes in SI joints in patients with early inflammatory back pain. Assessing bone marrow and/or subchondral bone enhancement suffices to detect inflammation. Inflammation in the joint space, the joint capsule and the ligaments hardly contributes to this detection, because it is associated with inflammation in the bone marrow and/or subchondral bone. About one-third of patients with recent-onset inflammatory back pain show inflammation, and about one-sixth of patients show structural changes in at least one SI joint, indicating that MRI might be a useful tool to diagnose sacroiliitis in patients with inflammatory back pain.

## Abbreviations

AS = ankylosing spondylitis; IBD = inflammatory bowel disease; MRI = magnetic resonance imaging; SE = spin echo; SI = sacroiliac; SpA = spondyloarthritis; STIR = short-tau inversion recovery.

## Competing interests

The authors declare that they have no competing interests.

## Authors' contributions

DvdH designed the study and performed statistical analysis. LHD collected data, read the MRI scans, and performed statistical analysis. RW read the MRI scans. RL performed statistical analysis. All authors interpreted the results, and wrote and commented on the manuscript.

## Figures and Tables

**Table 1:** Baseline characteristics of 68 patients with chronic inflammatory low back pain

Characteristic	All patients (*N *= 68)
Sex (% male)	38
Mean age (years) (SD)	34.9 (10.3)
Median symptom duration (months) (interquartile range)	18.0 [12.0–24.0]
Criteria for inflammatory low back pain	
Three criteria present (%)	56
Four criteria present (%)	41
Five criteria present (%)	3
Night pain present (%)	96^a^
HLA-B27 present (%)	46
History of inflammatory bowel disease present (%)	15
History of uveitis present (%)	15
History of psoriasis present (%)	24
Family history of ankylosing spondylitis present (%)	37

^a^Forty-five of the 47 patients in whom night pain was explored were reported confirmatory.

**Table 2:** Agreement between two observers with respect to magnetic resonance imaging (MRI) characteristics per site per sacroiliac joint in 68 patients with inflammatory low back pain

MRI characteristic per site	Sacroiliac joint	Present (*n*)	Absent (*n*)	Concordance rate	Discordance rate	Cohen's kappa value
Inflammation						
At any site	Right	18	40	0.85	0.15	0.68
	Left	14	39	0.78	0.22	0.51
Bone marrow ± subchondral bone	Right	17	43	0.88	0.12	0.73
	Left	12	47	0.87	0.13	0.65
Joint capsule	Right	5	54	0.87	0.13	0.46
	Left	3	47	0.74	0.26	0.12
Ligaments	Right	4	57	0.90	0.10	0.49
	Left	3	57	0.88	0.12	0.38
Joint space	Right	6	55	0.90	0.10	0.58
	Left	8	54	0.91	0.09	0.68
Chronic changes	Right	6	49	0.81	0.19	0.37
	Left	11	49	0.88	0.12	0.66

**Table 3:** Number of patients with abnormalities (inflammation, chronic changes or both) based on concordant observations by both readers

Abnormality	Number of patients with involvement of sacroiliac joints			
	
	Only the left sacroiliac joint	Only the right sacroiliac joint	Both sacroiliac joints	One or two sacroiliac joints
Inflammation	4	8	10	22
Structural changes (ankylosis, sclerosis, erosions)	5	0	6	11
Inflammation as well as structural changes	5	3	2	10
Inflammation				
Bone marrow or subchondral bone	4	9	8	21
Joint capsule	0	2	4	6
Ligaments	0	1	3	4
Joint space	4	2	4	10
